# Molecular Characterization and Pathogenicity of *Staphylococcus aureus* Isolated from Benin-City, Nigeria

**DOI:** 10.3390/microorganisms8060912

**Published:** 2020-06-16

**Authors:** Osahon Obasuyi, JoAnn McClure, Francis E. Oronsaye, John O. Akerele, John Conly, Kunyan Zhang

**Affiliations:** 1 Department of Pathology & Laboratory Medicine, University of Calgary, Calgary, AB T2N 4N1, Canada; osahon.obasuyi1@ucalgary.ca (O.O.); john.conly@albertahealthservices.ca (J.C.); 2 Department of Medical Laboratory Science, School of Basic Medical Sciences, College of Medical Sciences, University of Benin, Benin-City 300271, Nigeria; francisoronsaye@uniben.edu; 3 Department of Pharmaceutical Microbiology, Faculty of Pharmacy, University of Benin, Benin-City 300271, Nigeria; akerelej@uniben.edu; 4 Centre for Antimicrobial Resistance, Alberta Health Services/Alberta Public Laboratories/University of Calgary, Calgary, AB T2N 4N1, Canada; joannmarie.mcclure@albertahealthservices.ca; 5 Department of Microbiology, Immunology & Infectious Diseases, University of Calgary, Calgary, AB T2N 4N1, Canada; 6 Department of Medicine, University of Calgary, Calgary, AB T2N 4N1, Canada; 7 The Calvin, Phoebe and Joan Snyder Institute for Chronic Diseases, University of Calgary, Calgary, AB T2N 4N1, Canada

**Keywords:** MRSA, MSSA, molecular characterization, *C. elegans*, virulence, Nigeria

## Abstract

While numerous studies examine the epidemiology and molecular characterization of *Staphylococcus aureus* in most developed countries, the detailed molecular characterization and molecular epidemiology of *S. aureus* strains and clones in Africa is lacking. We determined the molecular epidemiology and virulence of 81 non-duplicate isolates of *S. aureus* from Benin-City, Nigeria, collected during January–July 2016, and compared with global strains. Forty-seven isolates (58.0%) were found to be methicillin-sensitive *Staphylococcus aureus* (MSSA), while 34 (42.0%) were methicillin-resistant *Staphylococcus aureus* (MRSA). ST152-MSSA (24.7%) and ST7-MRSA-V (19.8%) were the dominant groups identified, which were not genetically related to global predominant strains, but rather exhibited regional dominance. An interesting finding of the study was the presence of highly related strains in the region, which differed primarily in their methicillin resistance gene carriage, staphylococcal cassette chromosome *mec* (SCC*mec*), with 99.4–99.7% relatedness between the genomes of the strains within the MRSA–MSSA pairs. This suggests that the strains within a pair are experiencing gain or loss of SCC*mec* within local conditions, with evolution continuing to diversify the strains to a small degree. This study represents the most comprehensive genetic and virulence study of *S. aureus* in Nigeria.

## 1. Introduction

*Staphylococcus aureus* (SA) is an important human pathogen that causes a wide range of clinical infections, ranging from skin and soft tissue infection, to bacteremia and endocarditis [1]. Methicillin-resistant SA (MRSA) has become a leading cause of hospital-acquired infections worldwide, and accounts for the majority of *S. aureus* isolates in hospitals in Canada and the United States [2,3,4,5,6,7]. Established risk factors for hospital-acquired (HA) MRSA infections include recent hospitalization or surgery, residence in a long-term care facility, dialysis, and indwelling percutaneous medical devices and catheters [8]. Strains of MRSA have gone on to cause serious infections in community settings, affecting healthy children, athletes, and other individuals lacking typical risk factors for nosocomial MRSA acquisition [9,10,11,12,13,14,15,16,17,18,19,20,21,22]. These community-associated (CA) MRSA are generally susceptible to non β-lactam antibiotics and often carry the smaller type IV staphylococcal cassette chromosome (SCC) *mec* [23].

While numerous studies examine the epidemiology and molecular characterization of *S. aureus* in North America, Europe, and Asia, the detailed molecular characterization and epidemiology of *S. aureus* strains and clones in Africa is lacking. Studies have shown a higher incidence of *S. aureus* infection in Africa than other industrialized countries, and data have suggested that the prevalence of MRSA is increasing in many African countries [24,25,26]. It has also been shown that Panton–Valentine leukocidin (PVL) rates are high in both MRSA and methicillin-sensitive SA (MSSA) isolates in Africa; however, there is a lack of systematic reports containing detailed molecular and/or virulence determinant information [27,28,29,30]. As with the rest of Africa, information on *S. aureus* in Nigeria (in both health-care and community settings) is limited, with incomplete molecular information available [27,31,32,33,34,35,36,37,38,39]. Additionally, little is known about antiseptic susceptibility and the distribution of related resistance genes, which is of significance as there is a downward trend in the proper use of disinfectants and antiseptics in hospitals, resulting in disinfectant failures [40]. Given the lack of comprehensive data, the aim of this study was to determine the molecular epidemiology and virulence of *S. aureus* from Benin-City, Nigeria, and compare the genetic relatedness of these strains with global isolates. The results of this study could facilitate implementation of strategies for the prevention and effective management of staphylococcal infections in Nigeria.

## 2. Materials and Methods

### 2.1. Bacterial Strains and Isolate Collection

A total of 81 non-duplicate *S. aureus* isolates from various sources (urine, blood, semen, endo-cervix, vaginal, wound, aspirates, and so on) were obtained between January and July 2016. Clinical isolates were obtained from patients visiting two health facilities in Benin-City, Nigeria: the University of Benin-Health Centre and the University of Benin Teaching Hospital (UBTH). Written, informed consent was obtained from all participating patients. Canadian epidemic MRSA reference strains CMRSA1–10 were provided by the National Microbiology Laboratory, Health Canada, Winnipeg, Manitoba, Canada, and used for by pulsed field gel electrophoresis (PFGE) and genetic comparison. Another set of PFGE and genetic comparison reference strains, NRS382 (USA100), NRS383, NRS384, NRS123, NRS385, NRS386, and NRS387, for USA100-800, respectively, as well as the *C. elegans* control strain NCTC8325, were obtained through the Network on Antimicrobial Resistance in *S. aureus* (NARSA). *C. elegans* control strain M92 was provided by Dr. T. Louie from the University of Calgary, Canada.

### 2.2. Phenotypic Characterization and Antiseptic Gene Detection

Staphylococci were identified according to standard microbiological procedures, whereby isolates that were Gram-positive cocci (grape-like clusters), which produced catalase and positive to both slide and tube coagulase tests with human plasma, were considered as *S. aureus* [41]. Antibiotic susceptibility was determined using standard Clinical and Laboratory Standards Institute methods for antimicrobial disk diffusion using the following antibiotics: penicillin (10 IU), cefoxitin (30 µg), gentamicin (10 µg), erythromycin (15 µg), tetracycline (30 µg), doxycycline (30 µg), ciprofloxacin (5 µg), levofloxacin (5 µg), clindamycin (2 µg), trimethoprim/sulfamethoxazole (1.25/23.75 mcg), rifampin (5 µg), and linezolid (30 µg) (BD, Sparks, MD). Antiseptic resistance genes for chlorhexidine and quaternary ammonium compounds (*qacA*/*B*, *smr*), as well as mupirocin (both *mupA* and *mupB*) and methicillin resistance (*mecA*), were screened with a multiplex polymerase chain reaction (PCR) assay, as described [42]. DNA was extracted by rapid boiling method, as previously described [43].

### 2.3. Molecular and Genetic Characterization of Isolates

A multiplex PCR assay was used to distinguish SA from coagulase negative staphylococci, while simultaneously distinguishing MRSA from MSSA and detecting the PVL genes, as described [44]. *Staphylococcus aureus* isolates were fingerprinted by pulsed field gel electrophoresis (PFGE) following digestion with SmaI, according to a standardized protocol [45]. PFGE-generated DNA fingerprints were analyzed with BioNumerics Ver. 6.6 (Applied Maths, Sint-Martens-Lattem, Belgium), using a position tolerance of 1.0 and an optimization of 1.0. Isolates were further characterized with staphylococcal protein A (*spa*) typing [46], SCC*mec* typing [47,48], accessory gene regulator (*agr*) typing [49], and multilocus sequence typing (MLST) [50]. Genome sequencing was done on representative strains with Illumina MiSeq technology (Illumina inc, San Diego, CA, USA), and long sequence reads for selected isolates were obtained with MinIOIN sequencing (Oxford Nanopore, Oxford, UK). Virulence genes were identified in representatives strains by PCR amplification as described, or using oriTfinder software analysis of whole genome sequence (WGS) when it was available [51,52]. Prophage identification and annotation was achieved using PHASTER software [53,54]. Genetic relatedness was calculated using in silico DNA–DNA hybridization using the online software GGDC 2.1 with formula 3 [55]. Formula 3 was chosen owing to the highly related nature of the isolates, all fully sequenced and sharing similar sized genomes. Blast ring images were generated using BRIG v0.95 [56].

### 2.4. Isolate Groupings and Classification

*S. aureus* isolates were grouped according to the following criteria: isolates that were identical in PFGE pattern, *agr* type, *spa* type, MLST, SCC*mec* type, and antibiotic susceptibility profile were considered the same clone. Clones belonged to the same virulence group in the *C. elegans* infection model. Clones were combined into larger strain groups, with all isolates in the strain sharing identical *agr* type, *spa* type, MLST, and SCC*mec* type, but differing in PFGE pattern. Strains were further combined into larger MLST groups, whereby isolates in the group shared identical ST types. Regardless of similarities, MRSA and MSSA were considered different strains in this study.

### 2.5. Virulence Assessment by C. elegans Infection Model

Virulence was assessed on representative isolates from each clone using the *C. elegans* infection model, using established techniques [52,57]. The protocol was modified, whereby Bristol N2 *C. elegans* nematodes were synchronized with bleach and eggs were allowed to hatch and grow to the L4 stage on Nematode growth media (NGM) plates inoculated with *Escherichia coli* OP50 [57]. Once at L4, nematodes were washed from the plate, washed once in M9 buffer, and approximately 30 nematodes were suspended in M9 buffer added to each assay plate. *S. aureus* strains 8325 and M92 were used as positive and negative controls, respectively, and survival was scored every 24 h for 5 days. *C. elegans* survival curves were generated using GraphPad Prism 7 (GraphPad Software, La Jolla, CA, USA). Killing rates were calibrated as (%death_Isolate_ − %death_M92(-ve control)_)/(%death_8325(+ve control)_ − %death_M92(-ve control)_), with mean killing rates determined as the mean of three to five experimental replicates. *S. aureus* isolates were assigned to low virulence (mean killing rate of 0–0.39), moderate virulence (mean killing rate of 0.4–0.69), and high virulence (mean killing rate of 0.7–1.0) groups.

### 2.6. Ethical Approval

Ethical approval for this study was obtained from the ethical committee of the Edo State Federal Ministry of Health (Ref. # HA.577.187) (December 3, 2015-May 4, 2018), and the Ethics Research Committee of the Faculty of Pharmacy, University of Benin, Nigeria (Ref. # EC/FP/018/27) (3 December 2015–14 June 2018).

### 2.7. Genome Accession Numbers

The chromosomal genome sequence data have been deposited at GenBank under the following accession numbers: NGA102 (CP051191), NGA76 (CP051479), NGA104a (CP051482), NGA66a (CP051483), NGA71 (CP051484), NGA84b (CP051165).

## 3. Results

### 3.1. Overall Epidemiology of S. aureus Isolates from Benin-City

A total of 81 non-duplicate *S. aureus* isolates collected from the University of Benin Health Centre and the University of Benin Teaching Hospital in Benin-City, Nigeria were characterized and are summarized in Table 1. Of the 81 isolates, 47 (58.0%) were found to be MSSA, while 34 (42.0%) were MRSA. Antibiotic susceptibility profiles were determined for all isolates and, in general, MSSA showed low levels of antibiotic resistance. None of the MSSA isolates were resistant to cefoxitin, gentamicin, rifampin, or linezolid, while 47 (100%) were resistant to penicillin; 3 (6.4%) to erythromycin; 26 (55.3%) to tetracycline; 1 (2.1%) each to doxycycline, ciprofloxacin, and levofloxacin; and 3 (6.4%) to trimethoprim-sulfamethoxazole. Intermediate resistance was noted in one (2.1%) isolate to each of erythromycin, tetracycline, clindamycin, and trimethoprim-sulfamethoxazole; four (8.5%) isolates to doxycycline; and two (4.3%) isolates to ciprofloxacin. In terms of antiseptic resistance, the gene coding for *smr* was found in two (5.9%) of the MSSA isolates, while none of them carried the genes for *qacA*/*B*, *mupA*, or *mupB*. In contrast to the MSSA, the MRSA isolates showed a much higher degree of antibiotic resistance. A total of 34 (100%) of the MRSA isolates were resistant penicillin and cefoxitin, while 22 (64.7%) were resistant to gentamicin, 9 (26.5%) to erythromycin, 31 (91.2%) to tetracycline, 2 (5.9%) to doxycycline, 28 (82.4%) to ciprofloxacin, 27 (79.4%) to levofloxacin, 7 (20.6%) to clindamycin, 13 (38.2%) to trimethoprim-sulfamethoxazole, 1 (2.9%) to rifampin, and 1 (2.9%) to linezolid. Intermediate resistance was seen in 1 (2.9%) isolate to each of gentamicin, ciprofloxacin, and levofloxacin; 3 (8.8%) isolates to erythromycin; 11 (32.4%) isolates to doxycycline; and 2 (5.9%) isolates to clindamycin. Three (6.4%) of the MRSA carried the *mupB* gene, but none carried the *qacA/B*, *smr*, or *mupA* genes. 

### 3.2. Molecular Diversity of S. aureus from Benin-City

**Figure 1:**
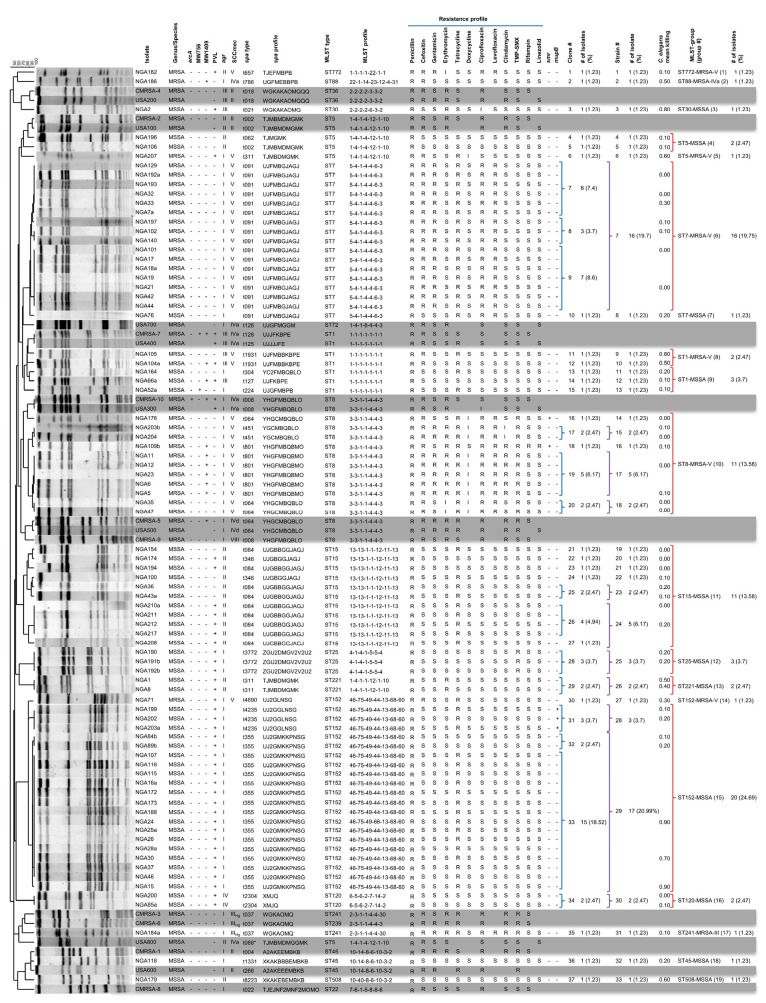
Genotypic and phenotypic characteristics of *Staphylococcus aureus* isolates from Benin-City, Nigeria. Molecular typing results showing the grouping of strains into clonal groups, strain groups, and MLST groups, with associated resistance profiles and *C. elegans* killing rates listed. Canadian (CMRSA1-10) and USA (USA100-800) epidemic control are included. *arcA*, arginine deaminase A; MW756, MW756 of genomic island νSa3-MW; MW1409, MW1409 of phage ϕSa2-MW; PVL, Panton–Valentine leukocidin; *agr*, accessory gene regulator; SCC*mec*, staphylococcal cassette chromosome *mec*; *spa,* staphylococcal protein A; MLST, multilocus sequence type; *smr*, small multidrug resistant; *mupB*, mupirocin; CD, calibrated death; MSSA, methicillin-susceptible *Staphylococcus aureus*; MRSA, methicillin-resistant *Staphylococcus aureus*; +, positive; −, negative; R, resistant; S, sensitive.

A more in-depth molecular characterization was done on the 81 *S. aureus* from Nigeria, with the results summarized in Figure 1. The isolates were grouped into 37 clones based on identical phenotypic and molecular characteristics. The clones were further grouped into 33 strains, with each strain sharing identical molecular characteristics, but differing by one to two bands in PFGE pattern. The strains were subsequently grouped into 19 MLST-groups, each sharing an identical ST type, but differing in other molecular characteristics. The most dominant clone observed was clone #33, comprising 15 (18.52%) isolates of ST152-t355-MSSA, which are PVL(+) and carry *agr* I. The second most prevalent clone was #9, with seven (8.6%) isolates of ST7-t091-MRSA-V, and *agr* I. The remaining clones were represented by one to six isolates each, as seen in Figure 1. For the most part, the same strain groupings existed as clone groupings, however, in some cases, clones that shared identical molecular typing, but differed by one to two PFGE bands were combined to form larger strain groups. Clones 32 and 33 were combined into strain #29 (ST152-t355-MSSA), representing the largest strain group with 17 (20.99%) isolates. Clones 7, 8, and 9 were combined into strain #7 (ST7-t091-MRSA-V), the second largest strain group with 16 (19.7%) isolates. Clones 26 and 27 were the final ones combined into strain #24 (ST15-t084-MSSA), comprising five (6.17%) isolates. Strains groups were further combined into MLST-groups, sharing identical MLST types, but differing in other molecular data. Strains 28 and 29 were combined into MLST group #15 (ST152-MSSA), the most commonly encountered one with 20 (24.69%) isolates. Strains 14, 15, 16, 17, and 18 were combined into MLST group #10 (ST8-MRSA), while strains 19, 20, 21, 22, 23, and 24 were combined into MLST group #11 (ST15-MSSA), each with 11 (13.58%) isolates. Also merged were strains 4 and 5 into MLST group #4 (ST5-MSSA); strains 9 and 10 into MLST group #8 (ST1-MRSA); and strains 11, 12, and 13 into MLST group #9 (ST1-MSSA).

**Figure 2:**
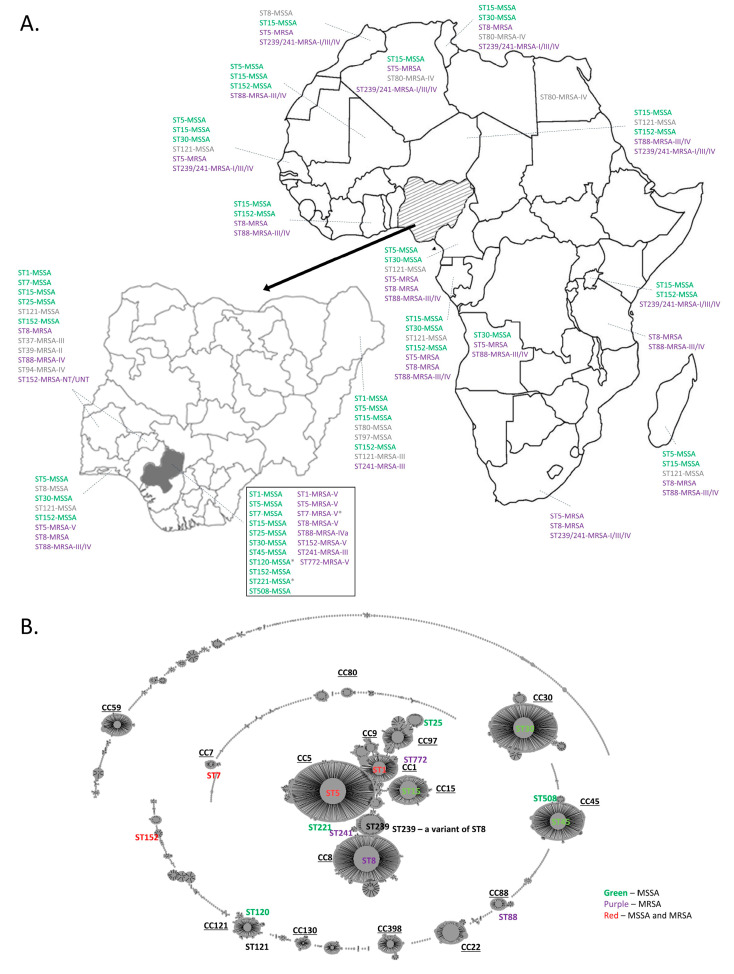
Global MRSA population snapshot. (**A**) MSSA/MRSA population structure, showing the major clones reported in each region of Africa along with the commonly associated SCC*mec* types (30). Nigeria (hashed and then enlarged), and Edo State (in dark grey) are also shown with their associated STs that have been reported. Strains detected in this study are indicated in the box. (**B**) Evolutionary relationships between the predominant MSSA/MRSA STs from our study are represented by e-BURST analysis (compared with the international MLST database, updated on 12 December 2018). Individual STs, as well as the clonal complexes to which they belong, are indicated. As in panel A, green (MSSA), purple (MRSA), and red (MSSA/MRSA).

The majority of MLST groups described in this study have previously been described in Nigeria and the rest of Africa, as shown in Figure 2A, which shows the major MRSA and MSSA clones identified in Africa, as well as a complete list of strain types reported in Nigeria. Strains identified in our study, but not previously reported in Nigeria or Africa include ST45-MSSA, ST120-MSSA, ST221-MSSA, and ST7-MRSA-V. Conversely, the majority of ST types previously described in Africa were found in this subset of isolates from Nigeria. These MLST groups are also well represented globally, as shown in Figure 2B, showing the evolutionary relationship of global MRSA.

Looking specifically at molecular factors, a total of 24 *spa* types were identified, with t355 being the most commonly found one among MSSA isolates, and t091 being the most common among MRSA. Eleven *spa* types (t657, t786, t4690, t1931, t801, t062, t4235, t346, t224, t1331, and t304) were identified for the first time in Nigeria. *agr* types for the *S. aureus* isolates were determined by PCR, with 58 (71.6%) found to belonged to *agr* I, 17 (21.0%) to *agr* II, 4 (4.9%) to *agr* III, and 2 (2.5%) to *agr* IV. SCC*mec* type V was identified in 32 (94.2%) MRSA isolates, while 1 (2.9%) MRSA isolate possessed each of the SCC*mec* type III and type IVa. Among the MSSA, 35 (74.5%) were PVL(+) and 10 (21.3%) were PVL(−), while only 2 (5.9%) MRSA were PVL(+) and 24 (70.6%) were PVL(−). One (1.23%) isolate carried the genomic island marker MW756 (associated with νSA3), while eight (9.9%) isolates carried the phage marker MW1409 (associated with ϕSa2-MW).

### 3.3. Antibiotic Resistance and Antiseptic Profiles among the S. aureus Groups

Antibiotic resistance profiles were determined with disc diffusion assays for the 81 *S. aureus* isolates and the results are summarized in Figure 1. Within the majority of MLST groups, strains were found to possess nearly identical antibiotic resistance profiles. The most notable exception was with resistance to tetracycline, which was found to differ in three of the MLST-groups. In group #9 (ST1-MSSA), two of the isolates were resistant to tetracycline, while one was sensitive. In group #11 (ST15-MSSA), nine of the isolates were tetracycline resistant, while two were sensitive. In group #15 (ST152-MSSA), 18 of the isolates were tetracycline resistant, while 2 were sensitive. No differences were seen in any other antibiotics within these groups. MLST group #10 (ST8-MRSA-V) was unusual in that a large degree of diversity was seen in the antibiotic resistance profiles of individual isolates. Of the 11 isolates in the MLST group, 7 were found to be resistant to gentamicin, 8 resistant to erythromycin (plus an additional 2 with intermediate resistance), 10 with intermediate resistance to doxycycline, 6 resistant to clindamycin (plus an additional 2 with intermediate resistance), 1 resistant to rifampin, and 1 resistant to linezolid. Similar to antibiotic resistance profiles, minimal differences were noted within an MLST group with respect to antiseptic resistance gene carriage. The exceptions were groups #15 (ST152-MSSA), which had three isolates that were *mupB*(+) and 17 *mupB*(−), and group #10 (ST8-MRSA-V), which had two isolates *smr*(+) and 9 *smr*(−).

### 3.4. S. aureus Groups Exhibited Varied Virulence Patterns

To assess virulence of the Nigerian *S. aureus*, representative isolates from each of the 37 clones were selected for testing in the *C. elegans* infection model. In clones with larger numbers of isolates, multiple representatives were selected, with the mean killing rates summarized in Figure 1. Isolates could be divided into three virulence groups: low virulence (mean killing rate of 0–0.39), moderate virulence (mean killing rate of 0.4–0.69), and high virulence (mean killing rate of 0.7–1.0) groups. With one exception, members of the same clone and/or same strain belonged to the same virulence group. Isolates in strain #29 (ST152-t355-MSSA) were the exception, whereby three isolates belonged to the high virulence group (mean killing rate of 0.7–0.9) and four belonged to the low virulence group (mean killing rates of 0.1 and 0.2). As with clone and strain groups, the majority of isolates within an MLST group possessed similar levels of virulence. As mentioned, MLST group #15 (ST152-MSSA) was an exception with low and high virulence representatives, as was MLST group #8 (ST1-MRSA-V), with both a high (mean killing rate of 0.8) and moderate (mean killing rate of 0.5) virulence representative. As a whole, only three MLST groups contained members that had high virulence, including groups #3 (ST30-MSSA), #8 (ST1-MRSA-V), and #15 (ST152-MSSA). Five MLST groups contained members with moderate *C. elegans* virulence, including groups #2 (ST88-MRSA-IVa), #5 (ST5-MRSA-V), #8 (ST1-MRSA-V), #13 (ST221-MSSA), and #19 (ST508-MSSA). Forty-four of the 53 isolates tested, representing 13 MLST groups, possessed low virulence in the *C. elegans* model, with mean killing rates ranging from 0.0 to 0.3.

Virulence gene carriage, as assessed by PCR amplification of 34 core virulence genes, did not reveal any patterns that could account for the observed killing rates among the groups (Table 2). All isolates tested were negative for the enterotoxin genes *sed*, *see*, *sei*, *sej*, *eta*, and *etb*, the fibrinogen binding protein gene *clfA*; and the elastin binding protein gene *ebps*. Conversely, all isolates were positive for the immunity evasive gene *scn*, the cytotoxin genes *hla* and *hld*, the fibronectin binding protein genes *fnbA* and *fnbB*, the MHCII analogue gene *map*, the intercellular adhesin gene *ica*, the protease gene *V8*, and the staphylokinase gene *sak*. Presence of the remaining genes varied between the MLST groups, as well as between the virulence groups.

### 3.5. Related MSSA and MRSA MLST Groups That Differed in Carriage of SCCmec

**Figure 3:**
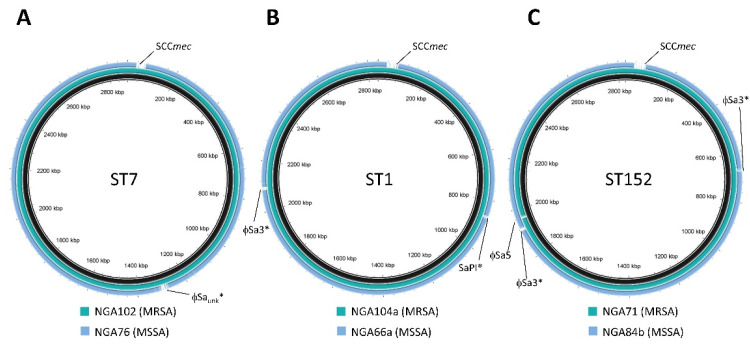
Relatedness of the MRSA and MSSA pair groups. Blast ring image generator (BRIG) analysis of the ST7 (**A**), ST 1 (**B**), and ST152 (**C**) pairs. In each case, a master sequence, containing all possible genomic features, is used as the reference and is shown in black. MRSA are represented by teal, and MSSA by blue. Discrepancies are labelled. *, the element is present in both the MRSA and MSSA strains, but differs in content/sequence between the two strains.

After detailed analysis of the MLST groups, we noted the presence of closely related pairs that appeared to only differ in their carriage of SCC*mec*. Group #7 (ST7-MSSA) and #6 (ST7-MRSA-V) represented one pair, while groups #8 (ST1-MRSA-V) and #9 (ST1-MSSA) represented a second pair, and groups #14 (ST152-MRSA-V) and #15 (ST152-MSSA) represented the third pair. Strains within each pair shared nearly identical molecular and virulence characteristics, differing in their resistance to methicillin, and having slight banding differences in their PFGE patterns (likely attributable to the SCC*mec* cassette). A representative from each group in the pairs was selected for whole genome sequencing in order to provide a more detailed comparison. Analysis using in silico genome to genome distance calculations revealed that NGA76 from group #7 (ST7-MSSA) and NGA102 from group #6 (ST7-MRSA-V) shared 99.7% relatedness. A comparison of 109 genetic traits, including 96 virulence genes, phage comparison, and antibiotic resistance, showed that the strains were identical in all but a few, primarily related to antibiotic resistance (see Supplementary Table S1). Of note, MSSA was missing the genes coding for penicillin-binding protein 2a (PBP2a), which was expected. A blast ring image generator (BRIG) analysis showing the similarity across the genomes is shown in Figure 3A. It indicates that the pair differ in two regions that are present in the MRSA and absent in the MSSA; that is, SCC*mec* near 40 Kbp and ϕSa_unk_ near 1320 Kbp. Strains NGA66a from group #9 (ST1-MSSA) and NGA104a from group #8 (ST1-MRSA-V) represent the second pair compared and found to share 99.5% relatedness by genome to genome distance calculator (GGDC) calculation. An examination of their virulence traits once again indicated that they differed only slightly, with notable variances being their *spa* types (UJFMBBKBPE for NGA104 vs. UJFKBPE for NGA66a), the lack of penicillin-binding protein 2a in MSSA, and the lack of *sek* and *seq* in MRSA (see Supplementary Table S1). BRIG analysis showed the presence of SCC*mec* in MRSA (near 40 Kbp) and its absence in MSSA (Figure 3B). It also indicated that, while pathogenicity island SaPI (near 860 Kbp) and ϕSa3 (near 2080 Kbp) are present in both strains, they differ in their content/structure. Strains NGA84b from group #15 (ST152-MSSA) and NGA71 from group #14 (ST152-MRSA-V) represent the third pair compared, and were found to share 99.4% relatedness by GGDC calculation. As with the previous pair, this pair differed by a few traits, with the biggest difference being *spa* type (UJ2GMKKPNSG for NGA84b vs. UJ2GLNSG for NGA71), the presence of penicillin-binding protein 2a in MRSA (see Supplementary Table S1), and the presence of ϕSa5 near 1940 Kbp in MSSA. They also differ in that ϕSa3 is located near 670 Kbp in strain NGS71, and near 1840 Kbp in strain NGS84b. BRIG analysis demonstrated these differences, with SCC*mec* at ~40 Kbp in MRSA and absent in MSSA, ϕSa5 present at 1940 Kbp in MSSA and absent in MRSA, as well as a difference in the content/structure of ϕSa3 near 680/1840 Kbp (Figure 3C).

## 4. Discussion

The molecular epidemiology of *Staphylococcus aureus* in Nigeria is not well described, with limited or incomplete data available from existing studies. Additionally, no data exist describing the virulence potential of representative clones from the country. To address this issue, we collected and typed 81 isolates of *S. aureus* from two health centers in Benin City in South-South Nigeria, representing the first time that comprehensive molecular and virulence characterization has been done on isolates from the region. In this study, eleven different MSSA (ST30, ST5, ST7, ST1, ST15, ST25, ST221, ST152, ST120, ST45, and ST508) and eight different MRSA (ST772, ST88, ST5, ST7, ST1, ST8, ST152, and ST241) MLST types were identified. ST152-MSSA and ST7-MRSA-V were the dominant methicillin-sensitive and methicillin-resistant MLST groups identified, accounting for 24.7 and 19.8% of isolates, respectively, while ST15-MSSA and ST8-MRSA-V were also highly represented, each accounting for 13.6% of the isolates (Figure 1). Previous studies characterizing *S. aureus* in Nigeria have identified all but three of the MSSA types that we detected, and all but one of the MRSA types, as shown in Figure 2A [30,31,32,33,35,36,38]. Identified for the first time in Nigeria were ST45-MSSA, ST120-MSSA, ST221-MSSA, and ST7-MRSA. The predominant MSSA in our study was PVL positive ST152-MSSA, a finding that was mirrored in a study by Okon et al., who found that the dominant MSSA clone detected in tertiary-care hospitals in North-East Nigeria was PVL positive ST152-MSSA [33]. Other studies have also reported this strain type as being widespread in African nations [28,29,30,38,58]. Our study differed from other studies in that the dominant MRSA identified in our region was ST7-MRSA-V (19.75% of the isolates), which has not been reported in the country. In 2009, Ghebremedhin et al. reported the presence of ST7-MSSA in 13% of the *S. aureus* they tested in South-West Nigeria; however, we only identified one ST7-MSSA isolate [32]. One can speculate that the ST7-MRSA evolved from these previously known ST7-MSSA, which had already proven to be well adjusted to conditions in the region, via acquisition of the SCC*mec* V cassette. SCC*mec* V is in fact commonly found in the region, occurring in six of our eight MRSA strain types. ST15-MSSA and ST8-MRSA represented the second most commonly encountered MSSA and MRSA, respectively, in our study and have been widely reported in Nigeria, with ST15-MSSA reported in the South-West and North-East regions of the country [32,36], ST8-MRSA in North-East Nigeria, and CC8-MRSA-III/IV/V in South-West Nigeria [31,33,38].

Expanding the analysis to Africa as a whole, we see that the major MRSA and MSSA strain types previously identified on the continent were also detected in our study and, conversely, that most of the strain types detected in our study have previously been described as occurring in Africa (Figure 2A). A comprehensive review detailing the top three MSSA and MRSA from various countries has shown that ST5-MSSA and ST15-MSSA were predominant in West Africa; while ST30, ST120/121, and ST152 were dominant in Central Africa; and ST88-MRSA was predominant in West, Central, and East Africa [30]. While ST15 and ST152 were among our most commonly isolated lineages, ST5-MSSA, ST30-MSSA, ST120-MSSA, and ST88-MRSA were infrequently encountered in our area, only accounting for 2.47%, 1.23%, 2.47%, and 1.23%, respectively. MRSA populations are dynamic, with a number of lineages evolving, and a select few becoming successful and predominating in each geographic location, although the precise reason remains unknown [59]. On a global scale, a comparison of our dominant MLST types for MSSA (ST152) and MRSA (ST7) to the major MLST types described worldwide shows that they are not genetically related to the predominant international ones (as shown by eBURST analysis in Figure 2B), but rather exhibit a regional dominance. CC152 strains are not commonly encountered and have been sporadically detected in some European countries and Australia, all carrying the PVL genes and devoid of most enterotoxin genes [60,61,62]. CC7-MRSA are also rare in other parts of the world, with isolates identified in Saxony and Australia [63].

A deeper examination of our isolates indicated that antibiotic resistance was high among them, a finding previously described in other studies from Nigeria, where high rates of resistance were previously noted for penicillin, trimethoprim-sulfamethoxazole, and tetracyclin, with MRSA also showing high rates of resistance to gentamicin, ciprofloxacin, erythromycin, and clindamycin [32,38]. Tetracycline (70.4% resistance) and penicillin (100% resistance) are used for a wide range of clinical applications, are cheap, are taken orally, and are sold in Nigeria without prescription. The same is true for fluoroquinolone antibiotics such as ciprofoloxacin (82.4% resistance) and levofloxacin (79.4% resistance), which are available as over-the-counter medications without prescription. With easy access to antibiotics and low levels of compliance with respect to dosing and duration of treatment, the high rates of resistance were not unexpected [64,65]. Antiseptic resistance, in contrast, was not particularly high in our isolates, with 5.9% of the MSSA carrying the *smr* gene, conferring resistance to monovalent cationic agents such as quaternary ammonium compounds and chlorhexidine, and 6.4% of the MRSA carrying the *mupB* gene, conferring resistance to mupirocin. There is no Nigerian reference with which to compare our antiseptic resistance results as, to date, no work has been done in this area. The appearance of resistance genes, however, could point to an emerging trend of disinfectant failure, likely connected to their improper usage, with guidelines not being followed for correct dilution, as well as to substandard products being manufactured and distributed [66]. Our data do suggest that antiseptic resistance surveillance may be needed in the region to determine if the resistance represents an emerging trend, or merely incidental gene carriage.

There are, likewise, no studies available to compare virulence gene carriage and *C. elegans* toxicity of our strains to previously reported strains in the region, country, or continent. We found no significant pattern of virulence gene carriage that could be associated with the observed toxicities in the *C. elegans* model, which ranged from low to moderate and high killing rates. *C. elegans* studies did reveal that three out of the seven (42.9%) isolates that were tested in the predominant MSSA group (ST152-MSSA) were associated with high virulence, ranging from 0.7 to 0.9. This, however, differed from the predominant MRSA group (ST7-MRSA-V), which was associated with low virulence. All seven of the ST7-MRSA-V isolates that were tested had virulence ranging from 0.0 to 0.3. Because both high and low virulence was noted in both MRSA and MSSA, this suggests that SA virulence in *C. elegans* might not be related to SCC*mec* carriage, although more studies are needed to support this claim. It is also important to reiterate that high virulence does not appear to be associated with strain predominance in this region, as ST30-MSSA (only representing 1.23% of isolates) and ST1-MRSA-V (representing 2.47% of isolates) were also high virulence strains. However, ST30-MSSA has been reported as being predominant in other parts of Nigeria and Africa [30,32], indicating that these high virulence strains may need to be monitored as they may in fact represent strains that will become predominant in this region of Nigeria as well.

An interesting finding of the study was the presence of highly related strains in the region, which differed primarily in their carriage of SCC*mec*. With 99.4–99.7% relatedness between the genomes of the strains within an MRSA–MSSA pair, they were shown to differ primarily in carriage of the methicillin resistance cassette. While the isolates are not identical to each other, as demonstrated with BRIG analysis, they differ almost exclusively in regions corresponding to mobile genetic elements, which are subject to significant evolutionary change. This suggests that the strains within a pair are experiencing gain or loss of SCC*mec* within local conditions, with evolution continuing to diversify the strains to a small degree. This has been suggested before with ST152-MSSA, which is a prevalent clone identified in west and central Africa [28,30,33,36,38,58,67], but where sporadic cases of ST152-MRSA have been noted in Nigeria [36,68]. It was suggested that these ST152-MRSA originated from an endemic ST152-MSSA via acquisition of the SCC*mec* cassette [27]. Our study similarly indicates that the ST152-MSSA is predominant, with only one ST152-MRSA identified. While the strain may have acquired a SCC*mec* cassette, ecological pressures appear to favor the MSSA, so it is not maintained. The ST7 group, on the other hand, may represent a situation where acquisition of SCC*mec* is favored by ecological pressures in the region. In Nigeria, ST7-MSSA has previously been described [32], but not ST7-MRSA. In our study, we identified predominantly ST7-MRSA, suggesting that antibiotic pressures created by easy access and low compliance favor carriage of SCC*mec*, meaning strains that have lost the resistance cassette (or alternatively strains that have not acquired it) are selected against. Finally, the ST1 group appears to represent a group where gain or loss of SCC*mec* is equally favored. In Nigeria, ST1-MSSA has been described [32,38], but in our region, MRSA and MSSA appear to be equally represented. Whether the cassette was acquired or lost, it appeared to be equally successful based on our results, although with such a low number of ST1 isolates detected that it is difficult to say with certainty. Further studies would be required to determine exactly to what degree antibiotic selective pressures impact the maintenance or loss of the SCC*mec* cassette.

## 5. Conclusions

This study represents the most comprehensive genetic and virulence study of *S. aureus* in Nigeria. While many of the strain types identified are common to Africa, we also noted some that showed a uniquely regional dominance, possibly highlighting evolutionary gain/loss of methicillin resistance within the region. This study was also the first to investigate antiseptic resistance in Nigeria and points to the need for further surveillance to determine if resistance is on the rise.

## Figures and Tables

**Table 1:** Distribution of *S. aureus* clinical isolates and their phenotypic characteristics.

	Isolates, #, (%)	Antibiotic Susceptibility # Isolates (%)													Antiseptic Resistance Gene (%)			
		Suscep.	PEN	FOX	GEN	ERY	TET	DOX	CIP	LVX	CLI	SXT	RIF	LZD	*qacA*/*B*	*smr*	*mupA*	*mupB*
*S. aureus* (81)	MSSA 47 (58.0)	R	47 (100)	0 (0)	0 (0)	3 (6.4)	26 (55.3)	1 (2.1)	1 (2.1)	1 (2.1)	0 (0)	3 (6.4)	0 (0)	0 (0)	0 (0.0)	2 (5.9)	0 (0.0)	0 (0.0)
		S	0 (0)	47 (100)	47 (100)	43 (91.5)	20 (42.6)	42 (89.4)	44 (93.6)	46 (97.9)	46 (97.9)	43 (91.5)	47 (100)	47 (100)				
		I	0 (0)	0 (0)	0 (0)	1 (2.1)	1 (2.1)	4 (8.5)	2 (4.3)	0 (0)	1 (2.1)	1 (2.1)	0 (0)	0 (0)				
	MRSA 34 (42.0)	R	34 (100)	34 (100)	22 (64.7)	9 (26.5)	31 (91.2)	2 (5.9)	28 (82.4)	27 (79.4)	7 (20.6)	13 (38.2)	1 (2.9)	1 (2.9)	0 (0.0)	0 (0.0)	0 (0.0)	3 (6.4)
		S	0 (0)	0 (0)	11 (32.4)	22 (64.7)	3 (8.8)	21 (61.8)	5 (14.7)	6 (17.7)	25 (73.5)	21 (61.8)	33 (97.1)	33 (97.1)				
		I	0 (0)	0 (0)	1 (2.9)	3 (8.8)	0 (0)	11 (32.4)	1 (2.9)	1 (2.9)	2 (5.9)	0 (0)	0 (0)	0 (0)				

Note: # = number; Suscep.: Susceptibility; PEN: Penicillin; FOX: Cefoxitin; GEN: Gentamicin; ERY: Erythromycin; TET: Tetracyclin; DOX: Doxycycline; CIP: Ciprofloxacin; LVX: Levofloxacin; CLI: Clindamycin; SXT: Trimethoprim-sulfamethoxazole; RIF: Rifampin; LZD: Linezolid; R: resistant; S: sensitive; I: intermediate resistance; *qacA/B*, *smr*: efflux mediated antiseptic resistance genes; *mupA*: mupirocin resistance gene A; *mupB*: mupirocin resistance gene B. MSSA, methicillin-sensitive *Staphylococcus aureus*; MRSA, methicillin-resistant *Staphylococcus aureus.*

**Table 2:** Virulence gene profiles for studied multilocus sequence typing (MLST) groups. Representatives from each MLST groups were selected and the presence of 34 virulence genes was determined by polymerase chain reaction (PCR) amplification.

		High Virulence				Moderate Virulence								Low Virulence									
		Virulence Gene	ST152-MSSA	ST30-MSSA	ST1-MRSA-V	ST5-MRSA-V	ST508-MSSA	ST221-MSSA	ST88-MRSA-IVa	ST1-MRSA-V	ST152-MRSA-V	ST7-MRSA-V	ST152-MSSA	ST7-MSSA	ST25-MSSA	ST45-MSSA	ST772-MRSA-V	ST5-MSSA	ST1-MSSA	ST8-MRSA-V	ST241-MRSA-III	ST15-MSSA	ST120-MSSA
Mean killing rate			0.9	0.8	0.8	0.6	0.6	0.5	0.5	0.5	0.3	0.3	0.2	0.2	0.2	0.2	0.1	0.1	0.1	0.1	0.1	0.0	0.0
**Exotoxin genes**	**Enterotoxin**	**sed**	**-**	**-**	**-**	**-**	**-**	**-**	**-**	**-**	**-**	**-**	**-**	**-**	**-**	**-**	**-**	**-**	**-**	**-**	**-**	**-**	**-**
		*see*	-	-	-	-	-	-	-	-	-	-	-	-	-	-	-	-	-	-	-	-	-
		*sei*	-	-	-	-	-	-	-	-	-	-	-	-	-	-	-	-	-	-	-	-	-
		*sej*	-	-	-	-	-	-	-	-	-	-	-	-	-	-	-	-	-	-	-	-	-
		*eta*	-	-	-	-	-	-	-	-	-	-	-	-	-	-	-	-	-	-	-	-	-
		*etb*	-	-	-	-	-	-	-	-	-	-	-	-	-	-	-	-	-	-	-	-	-
		*seb*	-	-	-	-	-	-	-	-	-	-	-	-	-	-	-	-	-	+	-	-	+
		*sec*	-	-	-	-	+	-	-	-	-	-	-	-	-	+	+	-	+	-	+	-	-
		*sea*	-	-	-	+	-	+	-	-	-	+	-	+	-	-	-	-	-	-	-	-	-
		*seg*	-	+	-	+	+	+	-	-	-	-	-	-	+	+	+	+	-	-	-	-	+
		*seh*	-	-	+	+	-	+	-	+	-	-	-	-	-	-	-	-	+	-	-	+	-
		*sek*	-	-	-	-	-	-	-	-	-	-	-	-	-	-	-	-	+	+	-	+	+
		*seq*	-	-	-	-	-	-	-	-	-	-	-	-	-	-	-	-	+	+	-	+	+
	Toxic shock	*tst*	-	-	-	-	+	-	-	-	-	-	-	-	-	+	-	-	-	-	-	-	-
	Immunity evasive	*chp*	-	-	+	+	+	+	+	+	-	-	-	-	+	+	-	-	+	-	-	+	-
		*scn*	+	+	+	+	+	+	+	+	+	+	+	+	+	+	+	+	+	+	+	+	+
	Cytotoxin	*hla*	+	+	+	+	+	+	+	+	+	+	+	+	+	+	+	+	+	+	+	+	+
		***hlb***	+	+	+	+	-	+	+	+	+	+	+	+	+	-	-	+	+	+	+	+	+
		***hld***	+	+	+	+	+	+	+	+	+	+	+	+	+	+	+	+	+	+	+	+	+
		***hlg***	-	+	+	+	+	+	+	+	-	+	-	+	+	+	+	+	+	+	+	+	-
**Adhesin genes**	**Fibrinogen binding**	***clfA***	-	-	-	-	-	-	-	-	-	-	-	-	-	-	-	-	-	-	-	-	-
	**Fibronectin binding**	***fnbA***	+	+	+	+	+	+	+	+	+	+	+	+	+	+	+	+	+	+	+	+	+
		***fnbB***	+	+	+	+	+	+	+	+	+	+	+	+	+	+	+	+	+	+	+	+	+
	**Collagen binding**	***cna***	+	+	-	-	+	-	+	-	+	-	+	-	-	+	+	-	+	+	+	+	+
	**Fibrinogen and bone binding**	***sdrC***	+	+	+	+	+	+	+	+	+	+	+	+	+	+	+	+	+	+	+	+	+
		***sdrD***	+	+	+	+	+	+	+	+	+	+	+	+	+	+	+	+	+	+	+	+	+
		***sdrE***	+	-	+	-	+	+	+	+	-	+	-	+	+	+	+	+	+	+	+	+	+
		***bbp***	+	+	-	-	-	-	-	-	-	+	+	+	-	-	-	-	-	-	-	-	+
	**Elastin binding**	***ebps***	-	-	-	-	-	-	-	-	-	-	-	-	-	-	-	-	-	-	-	-	-
	**MHCII analogue**	***map***	+	+	+	+	+	+	+	+	+	+	+	+	+	+	+	+	+	+	+	+	+
	**Intercellular adhesion**	***ica***	+	+	+	+	+	+	+	+	+	+	+	+	+	+	+	+	+	+	+	+	+
**Exoenzyme**	**Protease**	***V8***	+	+	+	+	+	+	+	+	+	+	+	+	+	+	+	+	+	+	+	+	+
	**Hyaluronoate lyase**	***hysA***	-	-	-	-	-	+	-	-	-	+	-	+	-	-	-	+	+	-	-	-	-
	**Staphylokinase**	***sak***	+	+	+	+	+	+	+	+	+	+	+	+	+	+	+	+	+	+	+	+	+

Note: Exotoxin genes: *sea*/b/c/d/e/g/h/i/j, staphylococcal enterotoxin A/B/C/D/E/G/H/I/J/K/Q; *tst*, toxic shock syndrome toxin; *chp*, chemotaxis inhibitory protein; *scn*, staphylococcal complement inhibitory protein; *hla/d*, *α*- and δ-toxin; *hlb*, *β*-toxin; *hlg, γ*-toxin. Adhesin genes: *clfA*, clumping factor; *fnbA*, fibronectin adhesive molecule A; *fnbB*, fibronectin adhesive molecule B; *cna*, collagen adhesive molecule A/B; *sdrC/D/E*, putative adhesin; *bbp*, bone sialoprotien adhesin; *ebpS*, elastin adhesin; *map*, major histocompatibility complex class II analogue protein; *ica*, polysaccharide intercellular adhesin. Exoenzyme genes: *V8*, serine protease; *hysA*, hyaluronidase; *sak,* staphylokinase; +, positive; −, negative.

## Supplementary Materials

The following is available online at https://www.mdpi.com/2076-2607/8/6/912/s1, Table S1: 109 traits in the MRSA–MSSA pairs, including molecular characteristics, antibiotic resistance genes, prophage carriage, and virulence genes.

## Abbreviations

SA	*Staphylococcus aureus*
MRSA	methicillin-resistant *Staphylococcus aureus*
MSSA	methicillin-sensitive *Staphylococcus aureus*
SCC*mec*	staphylococcal cassette chromosome *mec*
SNP	single nucleotide polymorphism
HA	hospital acquired
CA	community associated
PVL	Panton–Valentine leucocidin
UBTH	University of Benin Teaching Hospital
*C. elegans*	*Caenorhabditis elegans*
PFGE	pulsed field gel electrophoresis
PCR	polymerase chain reaction
*spa*	staphylococcal protein A
*agr*	accessory gene regulator
MLST	multilocus sequence type
WGS	whole genome sequence
ST	sequence type
GGDC	genome to genome distance calculator
BRIG	blast ring image generator
CC	clonal complex
