# Supplementary material for: Molecular Characterization and Pathogenicity of Staphylococcus aureus Isolated from Benin-City, Nigeria
Source: Microorganisms. 2020 Jun 16;8(6):912. doi: 10.3390/microorganisms8060912 (PMC7356805; doi:10.3390/microorganisms8060912)
Supplement: Supplementary file 1 [file microorganisms-08-00912-s001.pdf]

Supplementary Table S1. 109 traits in the MRSA-MSSA pairs, including molecular characteristics, antibiotic resistance genes, prophage carriage, and virulence genes.

		ST7-MRSA-V	ST7-MSSA	ST1-MRSA-V	ST1-MSSA	ST152-MRSA-V	ST152-MSSA
		Clone #8 (NGA102)	Clone #10 (NGA76)	Clone #13 (NGA104a)	Clone #14 (NGA66a)	Clone #30 (NGA71)	Clone #32 (NGA84b)
**Molecular Characteristics**	PVL	-	-	+	+	+	+
	arcA	-	-	-	-	-	-
	SCC*mec* Type	V	-	V	-	V	-
	agr	I	I	III	III	I	I
	spa Type	t091	t091	t1931	t127	t4690	t355
	MLST Type	ST7	ST7	ST1	ST1	ST152	ST152
**Antibiotic resistance genes**	Membrane transporter	+	+	+	+	+	+
	Quinolone resistance protein	+	+	+	+	-	-
	Beta-lactamase	+	+	+	+	+	-
	Undecaprenyl pyrophosphate phosphatase	+	+	+	+	+	+
	Tetracycline resistance protein TetM	-	-	-	-	-	-
	Penicillin-binding protein 2'	+	-	+	-	+	-
	Fosfomycin resistance protein FosB	-	-	-	-	-	-
	MATE efflux family protein	+	+	-	-	-	-
	Aminoglycoside 3′ phosphotransferase	-	+	-	-	-	-
	N-acetyltransferase	+	-	-	-	-	-
	MFS family major facilitator transporter	-	-	-	-	+	+
**Prophage**	φSa1	+	+	-	-	-	-
	φSa2	-	-	+	+	+	+
	φSa3	+	+	+	+	+	+
	φSa5	-	-	-	-	-	+
	φSa9	+	+	-	-	-	-
C. elegans	Calibrated Death	0.1	0.2	0.5	0.1	0.3	0.1
	**Virulence genes**						
**Acyl carrier protein**	*acpXL*	+	+	+	+	+	+
**Adherence**	*map*	+	+	+	+	-	-
	sdrC	+	+	+	+	-	-

	sdrD	+	+	+	+	+	+
	sdrE	+	+	+	+	+	+
	bbp	-	-	-	-	-	-
	epbs	+	+	+	+	+	+
	clfA	-	-	+	+	-	-
	fnbB	+	+	+	+	+	+
	fnbA	+	+	+	+	+	+
	clfB	-	-	+	+	-	-
	icaR	+	+	+	+	+	+
	icaA	+	+	+	+	+	+
	icaD	+	+	+	+	+	+
	icaB	+	+	+	+	+	+
	icaC	+	+	+	+	+	+
	cna	-	-	-	+	+	-
**Antiphagocytosis**	*cap8A*	+	+	+	+	+	+
	cap8B	+	+	+	+	+	+
	cap8C	+	+	+	+	+	+
	cap8D	+	+	+	+	+	+
	cap8E	+	+	+	+	+	+
	cap8F	+	+	+	+	+	+
	cap8G	+	+	+	+	+	+
	cap8H	+	+	-	+	-	-
	cap8I	+	+	+	+	-	-
	cap8J	+	+	+	+	-	-
	cap8K	+	+	+	+	-	-
	cap8L	+	+	+	+	+	+
	cap8M	+	+	+	+	+	+
	cap8N	+	+	+	+	+	+
	cap8O	+	+	+	+	+	+
	cap8P	+	+	+	+	+	+
**Exoenzyme**	*coa*	+	+	+	+	+	+
	v8	+	+	+	+	-	-
	vWbp	+	+	+	+	+	+
	sak	+	+	+	+	+	+
	geh	+	+	+	+	+	+
	sspC	+	+	+	+	+	+
	sspB	+	+	+	+	+	+
	sspA	+	+	+	+	+	+

	aur	+	+	+	+	+	+
	hysA	+	+	+	+	+	+
**+Immune evasion**	*adsA*	+	+	+	+	+	+
	scn	+	+	+	+	+	+
	spa	+	+	+	+	+	+
	sbi	+	+	+	+	+	+
	scn	+	+	+	+	+	+
	chp	-	-	+	+	-	-
**+Iron uptake**	*isdD*	+	+	+	+	+	+
	isdF	+	+	+	+	+	+
	srtB	+	+	+	+	+	+
	isdB	+	+	+	+	+	+
	isdA	+	+	+	+	+	+
	isdC	+	+	+	+	+	+
	isdE	+	+	+	+	+	+
	isdG	+	+	+	+	+	+
**Secretion system**	*esxA*	+	+	+	+	+	+
	esaA	+	+	+	+	+	+
	essA	+	+	+	+	+	+
	esaB	+	+	+	+	+	+
	essB	+	+	+	+	+	+
	essC	+	+	+	+	+	+
	esaC	+	+	+	+	+	+
	esxB	+	+	+	+	+	+
**Stress protein**	*KatA*	+	+	+	+	+	+
	clpC	+	+	+	+	+	+
	clpP	+	+	+	+	+	+
**Cytotoxin**	*hly/hla*	+	+	+	+	+	+
	hld	+	+	+	+	+	+
	hlgA	+	+	+	+	+	+
	hlgC	+	+	+	+	+	+
	hlgB	+	+	+	+	+	+
	hlb	+	+	+	+	+	+
**Toxic shock**	*tst*	-	-	-	-	-	-
**Enterotoxin**	*seh*	-	-	+	+	-	-
	sei	-	-	-	-	-	-
	sej	-	-	-	-	-	-
	sek	-	-	-	+	-	-

	seq	-	-	-	+	-	-
	sea	+	+	-	-	-	-
	seb	-	-	-	-	-	-
	sec	-	-	-	-	-	-
	sed	-	-	-	-	-	-
	see	-	-	-	-	-	-
	seg	-	-	-	-	-	-
**Panton-Valentine leukocidin**	*lukF-PV*	+	+	+	+	+	+
	lukS-PV	-	-	+	+	+	+
**Exfoliative toxin**	*eta*	-	-	-	-	-	-
	etb	-	-	-	-	-	-
**Triacylglycerol lipase precursor**	*lip*	+	+	+	+	+	+

Note: +, strain contains the gene or element; -, strain does not contain the gene or element.
